# Hot topics on fecal microbiota transplantation for the treatment of inflammatory bowel disease

**DOI:** 10.3389/fmed.2022.1068567

**Published:** 2022-12-02

**Authors:** Xiaochen Zhang, Dai Ishikawa, Toshifumi Ohkusa, Shinji Fukuda, Akihito Nagahara

**Affiliations:** ^1^Department of Gastroenterology, Juntendo University School of Medicine, Tokyo, Japan; ^2^Department of Regenerative Microbiology, Juntendo University School of Medicine, Tokyo, Japan; ^3^Department of Microbiota Research, Juntendo University Graduate School of Medicine, Tokyo, Japan; ^4^Department of Gastroenterology and Hepatology, The Jikei University Kashiwa Hospital, Chiba, Japan

**Keywords:** fecal microbiota transplantation, inflammatory bowel disease, ulcerative colitis, Crohn’s disease, pouchitis

## Abstract

Inflammatory bowel disease (IBD) is a chronic intestinal mucosal inflammatory disease with complex etiology. Traditional anti-inflammatory treatment regimens have yielded unsatisfactory results. As research continues to deepen, it has been found that the gut microbiota of patients with IBD is generally altered. The presence of microorganisms in the human gastrointestinal tract is inextricably linked to the regulation of health and disease. Disruption of the microbiotic balance of microbiota in the gastrointestinal tract is called dysbiosis, which leads to disease. Therefore, in recent years, the exploration of therapeutic methods to restore the homeostasis of the gut microbiota has attracted attention. Moreover, the use of the well-established fecal microbiota transplantation (FMT) regimen for the treatment of *Clostridioides difficile* infection has attracted the interest of IBD researchers. Therefore, there are an increasing number of clinical studies regarding FMT for IBD treatment. However, a series of questions regarding FMT in the treatment of IBD warrants further investigation and discussion. By reviewing published studies, this review explored hot topics such as the efficacy, safety, and administration protocol flow of FMT in the treatment of IBD. Different administration protocols have generally shown reassuring results with significant efficacy and safety. However, the FMT treatment regimen needs to be further optimized. We believe that in the future, individual customized or standard FMT implementation will further enhance the relevance of FMT in the treatment of IBD.

## Introduction

A growing number of studies have suggested that the presence of microbes in the human gastrointestinal tract is inextricably linked to the regulation of health and disease. Gut microbes ferment food into absorbable metabolites, synthesize essential vitamins, regulate the immune system, and act as a barrier to protect the gastrointestinal tract. Disruption of the gut microbiota balance, called dysbiosis, can lead to disease (1).

Inflammatory bowel disease (IBD) is an intestinal disease characterized by chronic inflammation of the intestinal mucosa that is prone to relapse. Common clinical types mainly include ulcerative colitis (UC), Crohn’s disease (CD), and pouchitis. The etiology of IBD is complex and diverse, which may be related to multiple interactive influences, such as environmental, microbial, genetic, and immune factors (2, 3). Traditional IBD treatment regimens have primarily focused on reducing inflammation. Although this treatment regimen has been continuously developed and updated, there are still drawbacks, such as easy relapse, immune tolerance, and drug resistance (4). Therefore, researchers continue to explore more effective treatment measures. It is generally accepted that the gut microbiota of patients with IBD is altered (3). The exploration of therapeutics to restore gut microbiota homeostasis has gained attention in recent years because the qualitative and quantitative profiles of the gastrointestinal microbiota in patients with IBD vary significantly compared to healthy individuals. Fecal microbiota transplantation (FMT) is an advanced microbial therapy that restores the gut microbiota and corrects the dysbiosis of the microbiota by providing full-spectrum microorganisms of healthy individuals to the patient so that the patient can obtain a complete functional ecosystem (5). In the *Clostridioides difficile* infection (CDI) treatment guidelines published in the United States and Europe, it is stated that FMT is a strongly recommended regimen for CDI with multiple recurrences (6, 7), with an effective rate of 92% (8). FMT has been implemented in a variety of disease fields (9–11), especially in improving the response of anti-PD-1 immunotherapy in metastatic melanoma (12, 13). Openbiome (14), a non-profit organization in the United States, is committed to providing an internationally standardized public stool bank for microbial treatment of various diseases. This provides the basic guarantee for FMT treatment. However, the use of FMT for the treatment of IBD is still progressing toward clinical application. In this review, we summarized hot topics such as efficacy, safety, and implementation of FMT for the treatment of IBD.

## Efficacy

Since the two cases of using FMT to treat patients with UC in 1989 proved effective (15, 16), researchers have been increasingly enthusiastic about exploring the use of FMT for IBD treatment.

### Efficacy of fecal microbiota transplantation in ulcerative colitis therapy

To date, six double-blinded, randomized controlled trials (RCTs) on the efficacy of FMT-induced remission in UC have been published (Table 1; 17–22). Moayyedi et al. recruited 75 patients with mild-severe UC (38 received FMT and 37 received placebo) and demonstrated that patients who received fecal enemas from donors (24%) had significantly higher rates of clinical remission at week 7 than those in the placebo enema group (5%) (*p* = 0.03). Two years later, Paramsothy et al. reported the results of their study of 81 patients with mild-moderate UC. Forty-one patients were included in the FMT group and 40 in the placebo group. At week 8, steroid-free clinical and endoscopic remission were achieved in 11 (27%) patients, which was significantly higher than that in the control group (3 patients [8%]) (*p* = 0.021). In an article published in 2019, Costello et al. enrolled 73 mild-moderate UC patients (38 in the FMT group and 35 in the placebo group). At week 8, steroid-free clinical and endoscopic remission were achieved in 12 (32%) of them. The treatment effect was significantly better than that observed in the placebo group, with only three of the 35 with complete remission (*p* = 0.03). In 2021, Haifer et al. also published the results of a RCT. Of the 35 mild-moderate UC patients recruited, 15 received FMT and 20 received a placebo. At week 8, the expected steroid-free clinical and endoscopic remission were achieved in 53% (*n* = 8) of patients in the FMT group, a significantly higher rate of remission than that in the placebo group of 15% (*n* = 3) (*p* = 0.027). Although positive results continued to emerge, as early as 2015, Rossen et al. reported contrary results. In 48 patients with mild-moderate UC, only seven of 23 patients receiving FMT achieved clinical and endoscopic remission at week 12, and five of 25 patients receiving placebo achieved remission, a result that was not significantly different (*p* = 0.51). Moreover, Crothers et al. published the results of a study with a small sample size (*n* = 12) in 2021. In the 12th week, only two of six patients in the FMT group achieved steroid-free clinical remission, while none in the placebo group achieved remission. There was no significant difference between the two groups (*p* = 0.45).

**TABLE 1:** Efficacy of FMT on UC patients with six double-blind, randomized controlled trials.

References	Rossen et al. (17)	Moayyedi et al. (18)	Paramsothy et al. (19)	Costello et al. (20)	Haifer et al. (22)	Crothers et al. (21)
Number of patients	48 (FMT: 23, placebo: 25)	75 (FMT: 38, placebo: 37)	81 (FMT: 41, placebo: 40)	73 (FMT: 38, placebo: 35)	35 (FMT: 15, placebo: 20)	12 (FMT: 6, placebo: 6)
Patient criteria	Mild-moderate (11 ≥ SCCAI ≥ 4, MES ≥ 1)	Mild-severe (Mayo: 4–12, MES ≥ 1)	Mild-moderate (Mayo: 4–10, MES ≥ 1/PGA ≤ 2)	Mild-moderate (Mayo: 3–10, MES ≥ 2)	Mild-moderate (Mayo: 4–10, MES ≥ 1)	Mayo: 4–10, MES ≥ 1, RBC ≥ 1, SFS ≥ 1
Pre-treatment	Bowel lavage	None	Bowel lavage	Bowel lavage	Amoxicillin, doxycycline, and metronidazole.	Ciprofloxacin, metronidazole, and bowel lavage
Steroid	Concomitant (<10 mg)	Concomitant	Taper 2.5 mg/w to free	Taper 5 mg/w to free	Taper 2.5 mg/w to free	free
FMT	2 times	6 times	41 times	3 times	49 times	85 times
Donor	Single	Single	Multiple (3–7 donors)	Multiple (3–4 donors)	Single	Single
Stool	Fresh	Fresh/frozen	Frozen −80°C	Frozen −80°C	Lyophilized	Frozen −20°C
Primary endpoint (FMT vs. placebo)	CR + ER at week 12 30 vs. 20%, *p* = 0.51	CR + ER at week 7 24 vs. 5%, *p* = 0.03	CR + ER/Er at week 8 27 vs. 8%, *p* = 0.02	CR + ER at week 8 32 vs. 9%, *p* = 0.03	CR + ER/Er at week 8 53 vs. 15%, *p* = 0.027	CR at week 12 2/6 vs. 0/6, *p* = 0.45
Clinical remission (FMT vs. placebo)	30 vs. 32%, *p* = 1.0	24 vs. 5%, *p* = 0.03	44 vs. 20%, *p* = 0.02	47 vs. 17%, *p* = 0.01	73 vs. 25%, *p* = 0.0045	/

El Hage Chehade et al. (23) conducted a meta-analysis of the different results of six double-blinded RCTs. A total of 324 patients were included in the analysis, and 30.43% of patients treated with FMT achieved clinical and endoscopic remission, significantly higher than 9.82% of patients in the placebo group who achieved clinical and endoscopic remission (*p* < 0.00001). In another non-double-blinded RCT (24), 90% of patients in the FMT group achieved the primary endpoint at week 8, compared with 50% in the placebo group. Considering the published conclusions so far, we believe that the efficacy of FMT for UC treatment is excellent.

### Efficacy of fecal microbiota transplantation in Crohn’s disease therapy

Cohort studies showed that FMT for CD treatment is generally effective (25–29). However, a few reports also showed a less obvious effect (30, 31).

Currently, only one RCT study has evaluated the clinical effect of FMT in CD (32). In 2020, Sokol et al. published a multicenter, single-blinded RCT study. Twenty-one patients who achieved clinical remission after 3 weeks of prednisolone therapy were randomly assigned to the FMT or placebo groups. No patients in either the FMT or placebo groups achieved the primary outcome of successful gut colonization with the donor microbiota at 6 weeks. The steroid-free clinical remission rates in the FMT and placebo groups were 87.5 and 44.4% at week 10 and 50 and 33.3% at week 24, respectively. Both results were not statistically significant. In 2021, a meta-analysis of FMT for CD treatment reported that the pooled rate of clinical remission in patients with CD reached 0.62, and that of clinical response was 0.79 (33).

Because CD lesions extend into the small intestine, determining the treatment response is expected to be more challenging than for UC. Moreover, it is expected that the response to FMT treatment will differ depending on the site of the lesion and whether it is a small or large bowel type. The results of using FMT for the treatment of CD are still controversial; hence, more convincing RCT studies are required.

### Efficacy of fecal microbiota transplantation in pouchitis therapy

Pouchitis is the most common complication of ileal pouch-anal anastomosis for refractory UC, with an incidence of up to 80% at 30-year follow-up (34). Some reports showed that 80% (35) of patients and 44% (36) with pouchitis achieved clinical remission after receiving FMT. A case report (37) also showed that antibiotic-refractory pouchitis improved significantly after FMT and persisted for more than 6 months. However, some other reports showed that (38–42) the efficacy was not very satisfactory, and no patient achieved clinical remission. Moreover, a recent RCT (43) report showed that FMT was not associated with relapse-free survival of pouchitis. In summary, the current results of the use of FMT in treating pouchitis are not satisfactory. Therefore, well-designed controlled studies are further needed.

## Safety

For a new treatment regimen for IBD, the public is most concerned about safety and efficacy. Most patients experience only transient discomfort, such as diarrhea, abdominal pain, bloating, borborygmus, nausea, vomiting, and increase in C-reactive protein level (17, 21, 22, 25, 26, 29, 31, 32, 44–63), which are believed to be an immune response caused by the infused fecal microbiota. There are also a small number of patients who have narcolepsy, fatigue (61), skin pruritus (29, 52, 62), testicular pain, rectal abscess (18), perianal pain or fistula (26), blood in the stool (27, 57), herpes zoster (57), and other complaints (64). However, these symptoms have not been shown to be directly related to FMT. Serious adverse events of worsening colitis requiring colectomy and hospitalization have been reported in some patients (18–20, 22, 26, 30, 32, 45, 57, 65, 66). Some of these exacerbated conditions were observed in the placebo group, while those in the FMT group may have been associated with a change in treatment regimen or a disproportionate host immune response induced by the new microbiota of the incomplete mucosa and disease progression rather than FMT itself. In addition, the spread of infection is a problem that doctors are very concerned about. Cytomegalovirus infections (17, 67), and CDI (18, 51) have been reported in FMT for the treatment of IBD. However, Rossen et al. concluded that CMV infection was not associated with FMT because patients were randomly assigned to the placebo group (17). In addition, Suskind et al. speculated that *C. difficile* infection in two patients, which occurred 3 and 4 months after transplantation, may not be related to FMT because the feces used showed no abnormal results on microbiological examination (51). Some studies have also described the risk of bacteremia. However, most of the fever symptoms in patients suspected of bacteremia resolved spontaneously within a short period (17, 21, 25, 26, 28, 31, 45–47, 49, 50, 62, 63, 68–72). Blood cultures were used in some studies to test whether a patient had bacteremia but did not yield positive results (47, 50, 62). However, a report (73) described a patient with CD who had positive blood cultures for multidrug-sensitive *Escherichia coli* bacteremia after FMT. Moreover, Grewal et al. (66) reported a patient with UC progression and toxic megacolon after FMT, who died of sepsis after surgery. Although not treated for UC, in March 2020, the FDA issued a safety warning^1^ that two patients with CDI were infected with drug-resistant *Escherichia coli* as a result of FMT treatment, and one died due to bacteremia (74). Despite occasional infections, rigorous donor screening is believed to reduce the risk of bacteremia and infectious disease transmission to almost zero.

Small bowel perforation (17), obstruction (26), and aspiration pneumonia (27, 31) caused by improper handling of routes of administration in the upper gastrointestinal tract (nasogastric, nasoduodenal, or nasojejunal tube) and lower gastrointestinal tract (transendocopic enteral tubing) have also been reported. This has caused severe pneumonia and intestinal bleeding leading to the death of a patient (27). The occurrence of these adverse events makes every doctor distressed, and the operation regimen is constantly improving. Moreover, a recent meta-analysis article analyzed published RCTs using FMT for various diseases and no significant difference in the incidence of serious adverse events was observed between the FMT and placebo groups (75). This suggests that FMT is a safe treatment modality.

## Implementation

There is still no unified standard protocol of FMT. The protocol of FMT affects the efficacy, safety, and patient acceptance of the treatment.

### Dose intensity and antibiotic pre-treatment

Fecal microbiota transplantation attempts to reverse dysbiosis by colonizing patients with healthy microbiota. It is now known that a single FMT treatment can restore the abnormal microbiota environment in most patients with CDI for several years (76, 77). However, according to the current study results, the effect of administration intensity on efficacy in patients with IBD is unstable.

Published articles showing the effect of a single FMT administration on clinical outcomes are controversial (69, 78). In addition, the lack of a control group in these articles makes it impossible to rule out other factors that may have contributed to the biased results. However, Mocanu et al. statistically analyzed that repeated FMT administrations were higher than single administrations in both clinical response (70 vs. 53%) and clinical remission rates (43 vs. 30%) (79).

Some researchers have conducted some double-blinded RCTs on multiple administrations of FMT. In 2015, Moayyedi et al. (18) published an article involving six administrations of FMT per patient. The remission rate of patients in the FMT group was significantly higher than that in the placebo group, which led to interest in the negative results of a study involving two administrations published by Rossen et al. (17) in the same year. Were the negative results of Rossen et al. related to the frequency of FMT use? The study by Paramsothy et al. (19), Haifer et al. (22), and Crothers et al. (21) performed 41, 49, and 85 FMTs on each patient, respectively, and the effect of using FMT was significantly better in the FMT group than in the placebo group. However, in 2019, Costello et al. (20) used a similar FMT implementation protocol as Paramsothy et al. (19); however, they only performed three FMT administrations, obtaining similar clinical outcomes as Paramsothy’s 41-administration study. This result raises the question of if more than 40 administrations are meaningful. Furthermore, how many administrations can give the best results? In a subgroup analysis of the number of administrations by Paramsothy et al. the pooled proportion of patients with UC who received more than 10 administrations and achieved clinical remission was 49%, significantly higher than the remission rate (27%) for patients with UC who received fewer than 10 administrations (*p* = 0.001) (54). There have been reports that there was no significant difference in adverse events (both severe and common adverse events) between the FMT and placebo groups in RCT studies involving the use of either single or multiple FMT administrations (75). However, too many administrations of FMT will bring inconvenience and psychological burden to patients; therefore, getting the best therapeutic effect under the premise of the least number of administrations is a topic worthy of further study. To the best of our knowledge, in addition to the effectiveness of antibiotic cocktail therapy in the treatment of patients with UC (80, 81), recent studies have shown that pre-treatment with antibiotics prior to FMT can improve FMT treatment efficacy by aiding microbiota colonization (82). We have previously reported (53, 60, 83) a clinical remission rate of approximately 35% with combined antibiotic pretreatment prior to the use of a single FMT, which is higher than the clinical remission rate observed by using multiple FMTs as reported by Rossen et al. (30%) (17) and Moayyedi et al. (24%) (18). Moreover, a case report showed that patients with refractory CD who received a single dose of FMT after pre-treatment with antibiotics had significantly improved symptoms (84). More RCTs are needed to verify the potentiating ability of antibiotic pre-treatment on FMT.

### Route administration

**FIGURE 1:**
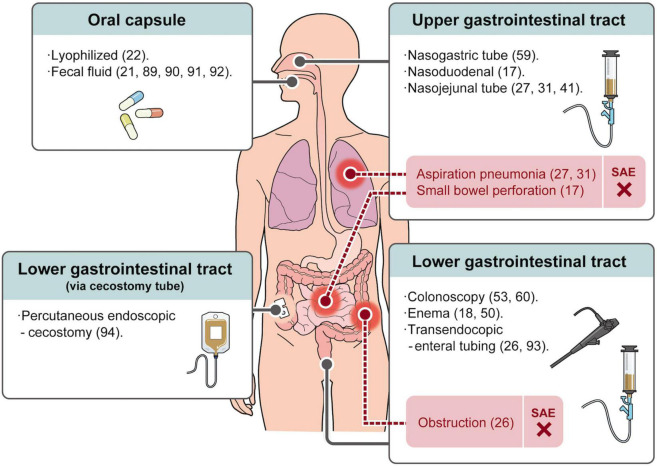
Route administration of FMT for IBD treatment and serious adverse events (SAE).

At present, the widely used FMT administration routes are mainly divided into upper gastrointestinal tract, lower gastrointestinal tract, and oral capsule-based FMT (Figure 1). There are meta-analysis statistics on the therapeutic effect of the FMT administration route on IBD, and the conclusions are inconsistent (54, 85). However, we believe it is challenging to assess the effect of the administration route on efficacy due to the use of different FMT protocols between studies. However, several routes of administration in the upper gastrointestinal tract (nasogastric, nasoduodenal, and nasojejunal tube) are inevitably affected by the distance from inflammation and the influence of proximal gastrointestinal secretions. Furthermore, in addition to the inherent risks of endoscopy, such as perforation, they may lead to symptoms such as aspiration pneumonia (31), vomiting (17, 31), runny nose, sore throat (59), and reflux (86).

Lower gastrointestinal administration routes mainly include enema and colonoscopy routes. Although patients can perform FMT with self-enema at home, possible related adverse events such as rectal abscess (18) and left-sided abdominal fullness (50) have been reported. The administration of FMT *via* colonoscopy has the advantage of transporting more stool to the site of inflammation (87). Moreover, it can detect the inflammatory state of the intestinal mucosa and compare the mucosal healing after treatment (88). However, frequent colonoscopies can also bring mental stress to patients. Therefore, an oral capsule-based FMT has recently attracted attention. In previous studies, oral capsule FMT was generally used as an adjunctive therapy (21, 89–91). A small sample-sized open-label study showed that oral capsule FMT can temporarily improve patients’ quality of life and reduce calprotectin (92). A double-blinded RCT (22) report in 2021 showed that oral lyophilized capsule FMT combined with antibiotic pre-treatment was significantly more effective than the placebo treatment (*p* = 0.027). In addition, no significant difference in FMT maintenance between an enema and oral capsule delivery was observed (91). Therefore, oral FMT capsules are a promising drug delivery option for long-term use to maintain a stable gut microbiota structure (21). More RCTs on oral capsule-administered FMT with high acceptability are required.

There are also some less frequently used methods, such as transendocopic enteral tubing (26, 93) and ercutaneous endoscopic cecostomy (94). It is also important to choose a method acceptable to the patient because patient compliance is the key to treatment.

### Treatment maintenance

Currently, the long-term maintenance effect of FMT in the treatment of IBD is unclear. Researchers have tried to maintain the diversity of gut microbiota through post-intervention of FMT to achieve the long-term efficacy of FMT in treating IBD. Repeating FMT several times after reaching remission to stabilize the intestinal environment is one method (95). An RCT study published in 2015 showed that of the nine patients who achieved clinical remission at week 7, eight were still in remission at week 52 with a monthly FMT interval (18). The article published by He et al. showed that the clinical remission rate (52%) after the initial FMT decreased slowly with the sustained remission rate after multiple FMT boosters, and 22.7% of patients were still in remission at 18 months (26). An RCT study randomly assigned 61 patients in remission to FMT to receive FMT or placebo administrations every 8 weeks for 48 weeks to determine the long-term maintenance effect of FMT. The results showed that FMT administration during the maintenance phase of UC patients could prolong the clinical, endoscopic, and histological remission of patients (96). It was further investigated that a second course of FMT consolidation therapy within 1 month could maintain the benefits of FMT in CD patients (65).

There are also attempts to maintain patient treatment outcomes in more light-hearted ways. For example, Wei et al. achieved the effect of slowing the loss of colonized microbiota by the oral administration of pectin that can be fermented into short-chain fatty acids and beneficial to intestinal microbiota (71). In our research group, we are conducting a double-blinded controlled RCT study to consolidate the efficacy of FMT in patients with UC by giving patients oral alginic acid (97). It is hoped that further research on the maintenance of efficacy will increase patients’ expectations and confidence in FMT for the treatment of IBD.

## Donor stool

The first major hurdle in FMT treatment is donor stool selection and preparation. Not only the transmission of pathogens can occur during FMT, as the impact of intestinal microbiota on patients with mental and endocrine diseases has been reported (9, 11). Hence, the screening of healthy fecal providers is currently a primary task. Many institutions also propose and continuously improve screening criteria according to the living background and the occurrence of epidemics in their respective regions (6, 14, 98, 99). Donor screening can be performed using questionnaires, blood tests, and stool tests. The basic questionnaire section should exclude infection risk factors such as HIV infection, exposure to viral hepatitis, high-risk sexual behavior, tattooing or piercing within 6 months, history of incarceration, travel history to areas endemic for infectious diseases, known history of infection, and risk factors for multi-drug resistant organisms. There are also potential microbiota-mediated conditions which should be determined, such as whether the donor has gastrointestinal disease, atopic disease, autoimmune disease, chronic pain syndrome, malignancy, and surgical history, and questions about the donor’s metabolic system, neurological system, mental, and medication conditions. Blood tests should mainly include complete blood count with differential, hepatic function, HIV, hepatitis, treponema pallidum, and parasite testing. Fecal testing should mainly include *C. difficile* toxin A/B, *Campylobacter*, *Salmonella*, *Shigella*, *Vibrio*, *Escherichia coli*, *Helicobacter pylori*, rotavirus, norovirus, adenovirus, COVID-19, and monkeypox. A more detailed screening should ensure patient safety but will reduce screening pass rates and increase screening costs. Therefore, maintaining a balance between the three methods is a question that needs to be considered. Of course, the relationship between FMT efficacy and donor feces is also a problem to be explored.

### Relationship between patients and donor

As far as we know, there are mainly two ways to obtain feces: one is from relatives or friends recommended by the patient and the other is from undirected stranger donors. Since some ethical, esthetic, and psychological barriers can be avoided by accepting stool from a donor recommended by the patient, the patient may be more receptive to the treatment. In addition, we previously reported higher long-term non-relapse rates for the treatment of UC with the stools of siblings compared to the stools of parents and offspring (*p* = 0.007) (60). The gut microbiota of siblings may be similar to the healthy microbiota state of the patient before IBD (100), and species originally present in the recipient’s microbiota are more likely to colonize the patient’s intestinal mucosa stably.

However, a meta-analysis showed no difference in the efficacy of feces from undirected stranger donors or patient-recommended donors for patients with CDI (101). Compared with patient-recommended donors, the undirected donor format has the advantages of avoiding screening time and starting treatment quickly, protecting the privacy of donor candidates, and saving costs for serving multiple patients after the successful screening. Therefore, doctors are more inclined to use the undirected donation of stranger feces.

### Fresh, frozen, or lyophilized stool

Using frozen stool can reduce the cost of FMT and increase the timeliness and safety of treatment. In addition, it has been reported that although freezing reduced the overall viability of the fecal microbiota by approximately 25%, the live microbiota composition was not significantly different from that of fresh feces (102). Cryopreservation of fecal samples for 6 months did not affect colony forming unit counts for some bacterial groups (*E. coli*, total coliforms, *Bifidobacteria*, total aerobes, *Lactobacilli*, or total anaerobic bacteria) (103). Therefore, frozen feces did not affect the efficacy of FMT in the treatment of CDI (103–105). However, there are meta-analysis statistics that the preservation status before FMT has an unstable impact on IBD (85, 106). UC patients treated with fresh donor stool had a lower pooled clinical remission rate (15%) than those with frozen stool (42%). Moreover, for CD patients, the remission rate for FMT with fresh stool was 36% higher than that with frozen stool (28%). Recently, the use of oral-fecal lyophilized capsules is a new method of drug delivery and storage. This delivery method requires that the capsules are always stored at −20°C and should not be directly transferred between refrigerators. If transfer is required, it should be kept on dry ice at all times to maintain the microbiota’s viability (21). It is difficult to link these three stool processes before drug delivery to IBD efficacy without RCTs that control for other potentially confounding variables.

### Donor microbiota characteristics

Donor biomarkers which are best for IBD have not been definitively reported. However, it has been reported that the microbial diversity of donor feces is associated with the efficacy of FMT in the treatment of IBD (31, 107). While testing the relationship between the abundance of the single donor’s gut microbial species and the therapeutic effect, some studies have also attempted to transplant the mixed feces of multiple people into the patient’s gut and achieved a significant effect compared to the placebo group (19, 20). However, there seems to be a super-donor phenomenon in the treatment of UC with FMT in previous studies. In 2015, Moayyedi et al. found that seven of nine patients with UC who achieved remission after FMT received stool from the same donor (18). Moreover, the efficacy rate of the multiple donors’ fecal microbiota transplant containing the donor number D054 was higher than that in patients who received multiple donors’ fecal transplant that did not contain the donor D054′s feces (*p* = 0.054) (19). From the current evidence, increasing the abundance of microbiota may not be the only condition for inducing remission. Further analysis of the study showed that a high abundance of specific species of *Bacteroides* (*B. fragilis* and *B. finegoldii*) in mixed donor feces was associated with the efficacy of FMT in patients with UC (108).

Fecal microbiota in patients with IBD is not only less diverse (109) but also often lacks commensal bacteria (110). For example, the bacterial phylum *Bacteroidota* (83, 111, 112), which produces zwitterionic capsular polysaccharides that suppress inflammation by regulating T cells, and *Bacillota*, which produces host-beneficial short chain fatty acids (SCFAs), are lacking. Therefore, some of the special bacteria carried in the guts of super-donors may colonize the guts of IBD patients if they supplemented the lost bacteria, and restoring the microbiota to a pre-morbid state could be beneficial. Reports showed that the presence of the bacterial genus *Ruminococcus* in the feces of the donors was associated with the induction of remission (18, 107). UC patients who achieved long-term FMT maintenance response showed a similar profile of microbiota to donors, especially *Bacteroidetes* species (60). In accordance with our previous report that dysbiosis of fecal microbiota in patients with UC is associated with loss of *Bacteroides* species diversity (83), we identified a relative abundance of 12 key *Bacteroidetes* species inversely associated with UC activity (112). The proportion of *Bacteroidetes* in feces was significantly increased in patients who underwent FMT (53). Therefore, the enrichment of *Bacteriodetes* in donor feces is one of our future research directions. In addition, different reports have shown that the intestinal microbiota of patients with CD has undergone inconsistent changes, such as a decrease of *Bacillota* (113), *Bididobacterium* (114), *Enterobacteriaceae* (115), or *Lactobacillus* (116), or an increase of *Helicobacter* species (117). In patients with UC and pouchitis, decreases of *Roseburia hominis* and *Faecalibacterium prausnitzii* (118) and absence of *Streptococcus* species (119) were also found. Therefore, determining the change of intestinal microbiota in IBD patients is a prerequisite for FMT treatment for IBD that cannot be ignored.

Although the results of the current study have not been able to establish the best donor guidelines for FMT, we can predict that in the future, the stool for the treatment of IBD will be selective and even customized.

## Conclusion

From the current research results, the effectiveness and safety of FMT in treating IBD are beyond doubt. However, the details of the entire execution process are still up for debate. New techniques for FMT are constantly being updated, and study has suggested that Sterile Fecal Filtrate Transfer (which only contains bacterial debris, proteins, antimicrobial compounds, metabolites, and oligonucleotides/DNA) can also eliminate symptoms and restore normal bowel habits in patients with CDI (120). It is unknown which substance in the gut produces this therapeutic effect. SCFA-producing bacteria are typically reduced in the gut of patients with IBD compared to healthy individuals (121). However, butyrate was increased in patients with UC who responded to FMT (122). Whether butyrate plays a major role in the treatment of FMT is unknown due to the lack of relevant clinical research data. Therefore, it is necessary to interpret the mechanism of FMT in the treatment of IBD from the perspectives of microbiology, immunology, and metabolism and propose a one-to-one customization scheme with a narrow-spectrum. Finally, while continuously optimizing the curative effect and maintaining the therapeutic outcome, it is essential to find the most acceptable route of administration for patients. In conclusion, more results from future studies are needed to obtain a perfect treatment of IBD using FMT.

## Author contributions

All authors contributed to the generation of the concept, wrote and edited the manuscript, and approved the submitted version.

## Footnotes

1
https://www.fda.gov/vaccines-blood-biologics/safety-availability-biologics/safety-alert-regarding-use-fecal-microbiota-transplantation-and-risk-serious-adverse-events-likely
